# Phenotypic Characterization of a Virulent PRRSV-1 Isolate in a Reproductive Model With and Without Prior Heterologous Modified Live PRRSV-1 Vaccination

**DOI:** 10.3389/fvets.2022.820233

**Published:** 2022-04-07

**Authors:** Heinrich Kreutzmann, Julia Stadler, Christian Knecht, Elena L. Sassu, Ursula Ruczizka, Yury Zablotski, Eleni Vatzia, Gyula Balka, Marianne Zaruba, Hann-Wei Chen, Christiane Riedel, Till Rümenapf, Andrea Ladinig

**Affiliations:** ^1^Department for Farm Animals and Veterinary Public Health, University Clinic for Swine, University of Veterinary Medicine Vienna, Vienna, Austria; ^2^Clinic for Swine, Centre for Clinical Veterinary Medicine, Ludwig-Maximilians-University Munich, Oberschleissheim, Germany; ^3^Department of Pathobiology, Institute of Immunology, University of Veterinary Medicine Vienna, Vienna, Austria; ^4^Department of Pathology, University of Veterinary Medicine Budapest, Budapest, Hungary; ^5^Department of Pathobiology, Institute of Virology, University of Veterinary Medicine Vienna, Vienna, Austria

**Keywords:** porcine reproductive and respiratory syndrome virus (PRRSV), experimental reproductive model, PRRSV-1, AUT15-33, PRRSV-1 phenotypic characterization, vaccine efficacy, ReproCyc® PRRS EU, modified live virus vaccine

## Abstract

Reproductive disorders induced by porcine reproductive and respiratory syndrome virus (PRRSV) cause high economic losses in the pig industry worldwide. In this study, we aimed to phenotypically characterize a virulent PRRSV-1 subtype 1 isolate (AUT15-33) in a reproductive model. Furthermore, the protective effect of a heterologous modified live virus vaccine (ReproCyc® PRRS EU) was evaluated. In addition, PRRSV AUT15-33 was genotypically compared to other well-characterized isolates. Sixteen gilts were equally divided into four groups: a vaccinated and infected group (V–I), a vaccinated and non-infected group (V–NI), a non-vaccinated and infected group (NV–I), and a non-vaccinated and non-infected (NV–NI) group. After PRRSV infection on gestation day 84, all gilts were clinically examined on a daily basis, and blood samples were taken at five timepoints. Necropsy was performed 3 weeks after infection. The fetal preservation status was assessed, and PRRSV RNA concentrations were measured in the blood and tissue samples from all gilts and fetuses. After infection, all four gilts in the NV–I group were viremic throughout 17 days post-infection (dpi), whereas two gilts in the V–I group were viremic at only one timepoint at 6 dpi. The viral load was significantly higher in gilt serum, tracheobronchial lymph nodes, uterine lymph nodes, maternal endometrium, and fetal placenta of NV–I gilts compared to the V–I ones (*p* < 0.05). Moreover, the preservation status of the fetuses derived from NV–I gilts was significantly impaired (55.9% of viable fetuses) compared to the other groups (*p* < 0.001). Upon comparison with other known isolates, the phylogenetic analyses revealed the closest relation to a well-characterized PRRSV-1 subtype 1 field isolate from Belgium. In conclusion, the high virulence of AUT15-33 was phenotypically confirmed in an experimental reproductive model. The vaccination of the gilts showed promising results in reducing viremia, fetal damage, and transplacental transmission of the PRRSV-1 strain characterized in this study.

## Introduction

Economically, the porcine reproductive and respiratory syndrome virus (PRRSV) is one of the most devastating viral pathogens in pig production, causing reproductive failure in pregnant gilts and sows and respiratory disease in pigs of different age groups (1). After an amendment of the arterivirus taxonomy, the current PRRSV species was divided into two genetically and antigenically distinct species: PRRSV-1 (the former European genotype 1) and PRRSV-2 (the former North American genotype 2) (2). According to the International Committee on Taxonomy of Viruses, these two species have been taxonomically classified into the species *Betaarterivirus suid 1* and *Betaarterivirus suid 2* (3). Based on the high genetic diversity, PRRSV-1 can be further divided into at least three subtypes: Pan-European subtype 1 and Eastern European subtypes 2 and 3 (4–6). Within these subtypes, a very high genetic diversity can be detected (7, 8).

The high genetic diversity of this RNA virus, together with the high mutation and recombination rate, can lead to the development of highly virulent strains that have mainly been described for PRRSV-2 in North America (9, 10) as well as in Asia (11, 12). Outbreaks with these virulent strains are mostly characterized by severe clinical signs (e.g., high fever, petechial hemorrhages, general illness, sow abortion and mortality syndrome) and higher mortality rates and viral loads in blood and tissues compared to the less virulent PRRSV (12, 13). Within PRRSV-1, virulent strains have traditionally been linked to subtype 3 strains (Lena and SU1-bel strains). However, more recently, variants causing virulent patterns have also been identified within subtype 1 in Belgium, Hungary, Italy, and Austria (13V091, 9625/2012, PR40/2014, and AUT15-33, respectively) (6, 14–16).

In the spring of 2015, a previously PRRSV-seronegative piglet-producing farm in Lower Austria experienced an outbreak with about 60% repeat breeding in sows and farrowing losses of up to 90%. From this first registered outbreak, the PRRSV-1 subtype 1 strain AUT15-33 was isolated and partially sequenced (GenBank accession numbers KT265737.1, KT265738.1, and KU494019.1). Since then, derivatives of AUT15-33 have been found in various farms in Austria and Germany (16), often associated with episodes of reproductive failure. The emergence of this virulent variant in Western Europe unsettled pig producers and raised concerns about the efficacy of currently licensed PRRSV vaccines.

Vaccination of sows is a widely applied strategy used to minimize the clinical and economic impact of PRRSV infections (17–19). Several modified live virus (MLV) and inactivated vaccines have been developed for the control of PRRSV. Commercial MLV vaccines have been effective at providing protection against homologous strains, whereas different levels of cross-protection after challenge with heterologous PRRSV strains can be observed (20–22). In 2015, a new, modified live PRRSV-1 vaccine for active immunization of breeding females was introduced to the European market (ReproCyc® PRRS EU, Boehringer Ingelheim Vetmedica GmbH, Germany) (23). The safety and protective efficacy of this vaccine have been demonstrated by a previous field study, with reduced mortality during the suckling period and improved growth performance of piglets before weaning being effects comparable to those of another commercial PRRSV-1 vaccine (24). However, no controlled experiment has been performed to date to evaluate the efficacy of this modified live PRRSV-1 vaccine against challenge with this particular PRRSV-1 strain in terms of *in utero* PRRSV transmission to piglets and reduction of fetal compromise and fetal death.

Therefore, the objective of the present study was first to characterize the virulence of AUT15-33 in a reproductive model under experimental conditions. Moreover, the protective efficacy of a PRRSV-1 modified live virus vaccine (ReproCyc® PRRS EU) upon challenge with this strain was evaluated.

## Materials and Methods

### Animals and Experimental Design

Sixteen clinically healthy gilts were purchased from a specialized gilt producer (PIC Deutschland GmbH) and housed in a piglet-producing farm in Lower Austria which was not suspected for PRRS based on routine serological monitoring. Serum samples were collected from all gilts after arrival at the farm to confirm the PRRSV-negative status by means of ELISA (IDEXX PRRS X3 Ab Test®, IDEXX Europe B.V., Hoofddorp, Netherlands) and PCR. According to the vaccination protocol of the farm, all gilts were vaccinated against porcine parvovirus in combination with erysipelas (Parvoruvac®, previously Merial GmbH, France; now Ceva Santé Animale, France) and against influenza virus (Respiporc FLU3®, IDT Biologika GmbH, Germany; now Ceva Santé Animale, France) twice before breeding (4 weeks and 4 days prior). In addition, all gilts were vaccinated against porcine circovirus type 2 (Ingelvac CircoFLEX®, Boehringer Ingelheim Vetmedica GmbH, Germany) once in mid-gestation.

**Figure 1:**
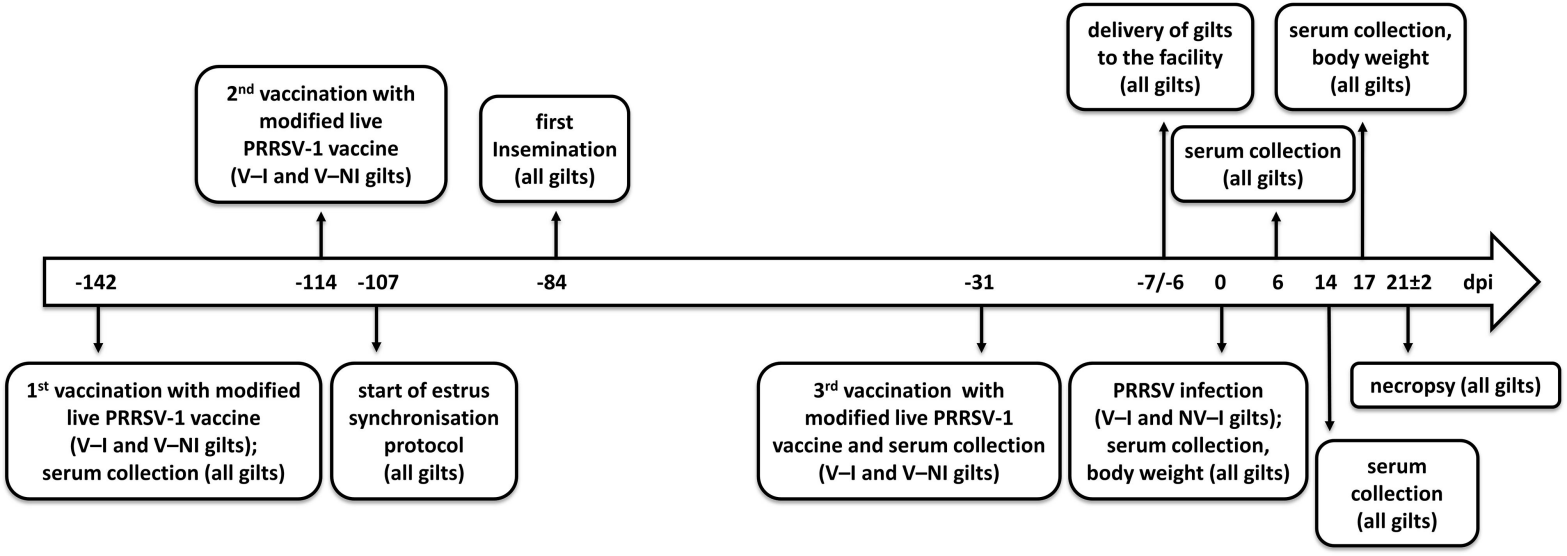
Timeline of events. Days post-infection (dpi) are indicated in the center of the arrow. Eight gilts (V–NI gilts, *n* = 4; V–I gilts, *n* = 4) were vaccinated with the modified live PRRSV-1 vaccine ReproCyc® PRRS EU twice before insemination and once in mid-gestation. Eight other gilts were left unvaccinated (NV–NI gilts, *n* = 4; NV–I gilts, *n* = 4). At 1 week before the challenge, the gilts were transported to the biosafety level 2 isolation unit of the University of Veterinary Medicine Vienna. After the challenge with PRRSV-1 strain AUT15-33 (only V–I and NV–I gilts), the samples were collected, the body weight was assessed, and necropsy was performed from all gilts (*n* = 16) at 3 weeks after the challenge of the V–I and NV–I gilts. NV–NI, non-vaccinated and non-infected gilts; V–NI, vaccinated and non-infected gilts; V–I, vaccinated and infected gilts; NV–I, non-vaccinated and infected gilts.

Half of the gilts (*n* = 8) were randomly selected and vaccinated with ReproCyc® PRRS EU intramuscularly twice before insemination (142 and 114 days prior to challenge) and once in mid-gestation (31 days prior to challenge) according to the manufacturer's instructions. The vaccinated gilts were housed in a separate facility in order to avoid vaccine virus transmission to the non-vaccinated gilts. Taking into account the strict biosecurity measures, the housing conditions, including the animal caretakers and the feed, were the same in both facilities. Before the last trimester of gestation (7 and 6 days prior to challenge), the gilts were transported to the biosafety level 2 (BSL-2) isolation unit of the University of Veterinary Medicine Vienna (Figure 1). The vaccinated and non-vaccinated animals were transported on different days to avoid cross-contamination. Upon arrival, both vaccinated and non-vaccinated gilts were clinically investigated and randomly split into two groups (NV–NI = non-vaccinated and non-infected: gilts 1, 2, 3, and 4; V–NI = vaccinated and non-infected: gilts 5, 6, 7, and 8; V–I = vaccinated and infected: gilts 13, 14, 15, and 16; NV–I = non-vaccinated and infected: gilts 21, 22, 23, and 24). Each of the four different treatment groups was housed in an individual room with separate air spaces. All gilts had free access to water and were fed *ad libitum* with a commercial diet for pregnant sows.

### PRRSV Isolate and Challenge

The PRRSV-1 isolate AUT15-33 was propagated on porcine alveolar macrophages (PAM) as previously described (16) for three passages to obtain 100 ml of virus stock (5.6 × 10^5^ TCID_50_/ml) for challenge and sequence determination. On gestation day 84, the V–I gilts (*n* = 4) and NV–I gilts (*n* = 4) were inoculated at a dose of 3 × 10^5^ TCID_50_ in a total volume of 4 ml; 2 ml was administered intramuscularly, and 1 ml was administered into each nostril.

### Determination of Full-Length Sequence

Twenty-five milliliters of cell culture supernatant from AUT15-33-infected PAM was subjected to low-speed centrifugation for 10 min at 10,000 rpm in a Fiberlite FS13 rotor to sediment cellular debris. The supernatant was passed through a 0.45-μm bottle top filter and concentrated by centrifugation in a Beckman Ti 55.2 rotor at 35,000 rpm for 2 h. The resulting sediment was resuspended in 1 ml of phosphate-buffered saline. The insoluble matter was removed by centrifugation at 10, 000 × *g* for 2 min, and virions were finally concentrated in a Beckman TLA45 rotor at 45,000 for 1 h. The virus-containing sediment was resuspended in 50 μl H_2_O and subjected to RNA preparation using the RNAeasy Mini Kit (QIAGEN GmbH, Hilden, Germany), yielding 2.3 μg RNA. Then, 500 ng of this RNA was either submitted to paired-end next-generation sequencing (Clontech Laboratories Inc., USA), yielding 2 × 10^6^ reads, or subjected to cDNA transcription using MuLV Reverse Transcriptase (New England Biolabs GmbH, Germany). The partially degenerated oligonucleotide primers facilitated the generation of a set of overlapping fragments with a size of 1.5 to 3 kb (Supplementary Table S1). The PCR products were subjected to Sanger sequencing using the same set of primers (Eurofins Genomics GmbH, Germany). The 5′ end was determined by 5′ RACE. To this end, the first-strand cDNA was synthesized from total RNA using the primer PRS338 (TGGTCRGACACGTGCATGGAG; position, nt 650–670) and purified using PCR Kleen™ spin columns (Bio-Rad Laboratories, CA, USA). The cDNA was poly-A tailed with terminal deoxytransferase and 1 mM ATP (New England Biolabs). The reaction was terminated by heating to 65°C and subjected to a semi-nested PCR using primer PRS324 (CAATGGCACCAAGGTCAGTGTCC; position, nt 341–363), oligodT, and OneTaq polymerase (New England Biolabs). The resulting PCR product of 363 nt was ligated to a pGEM-T vector and transformed in *Escherichia coli* DH5a. Eight clones were sequenced, and the consensus was considered as 5′ end. From both sequencing approaches, a consensus sequence was assembled. The full genomic sequence has been submitted to GenBank (accession number MT000052).

### Nucleotide Sequence Comparisons

**Figure 2:**
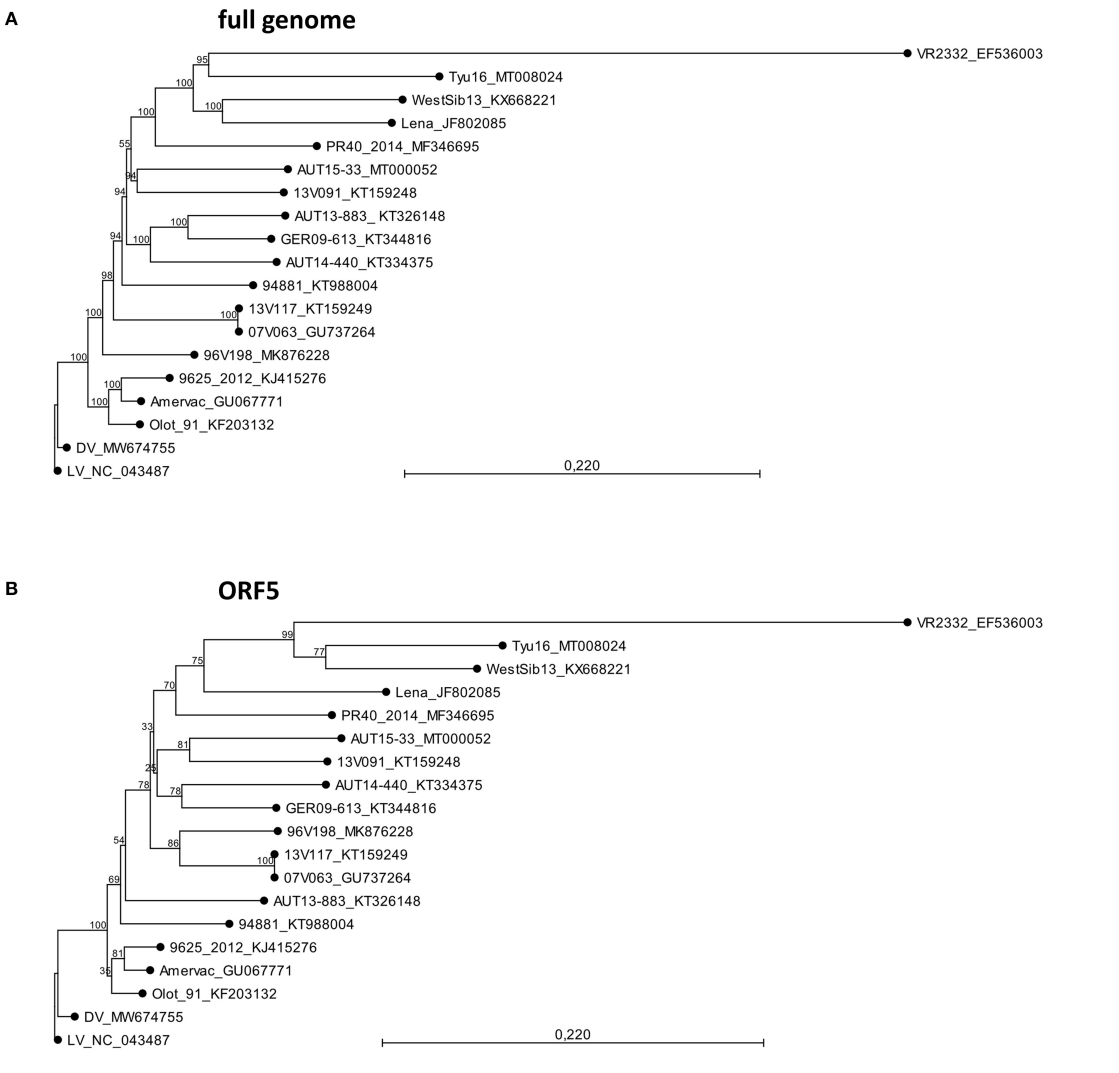
Phylogeny calculations with selected porcine reproductive and respiratory syndrome virus strains for the full genome **(A)** and ORF5 sequences **(B)**. The phylogenetic trees were calculated in CLC workbench package by neighbor joining method with a Kimura 80 algorithm. The bootstrap analysis was set to 1,000 replicates. Both phylogenetic trees are rooted to LV. The scale bar indicates the number of nucleotide substitutions per site.

A sequence comparison of all deposited full-length genomes of PRRSV-1 and PRRSV-2 was performed using BLAST (25). AUT15-33 was then compared with PRRSV-1 subtype 1 strains for which both virulence and whole-genome sequence data have been published (Table 1). These include the Spanish strain Olot/91 (accession number KF203132), the Belgian strains 07V063, 13V091, and 13V117 (GU737264, KT159248, and KT159249), the German strain GER09-613 (KT344816), the Hungarian strain 9625/2012 (KJ415276), the Italian strain PR40_2014 (MF346695), and the Austrian strains AUT13-883 and AUT14-440 (6, 14, 15, 30, 32, 33, 40). PRRSV-1 subtype 1 vaccine strains were included if available in GenBank (KT988004, GU067771, MW674755, and MK876228). Reference strains for PRRSV-1 subtype 1 Lelystad virus (LV; NC_043487), subtype 2 WestSib (KX668221), subtype 3 Lena (JF802085), a non-subtypeable PRRSV-1 strain Tyu16 (MT008024), and PRRSV-2 prototype VR2232 (EF536003) were added as well (5, 13, 27, 28, 35–37). A phylogeny calculation of these strains was performed for the full genome sequence (Figure 2A), and ORF5 (Figure 2B) was calculated in CLC workbench package by neighbor joining method with a Kimura 80 algorithm (QIAGEN Aarhus A/S, Aarhus, Denmark). The bootstrap analysis was set to 1,000 replicates, and both phylogenetic trees were rooted to LV.

**Table 1:** Overview of the nucleotide lengths for individual open reading frames of selected porcine reproductive and respiratory syndrome virus isolates.

	**PRRSV-1 subtype**	**Year of isolation**	**Outlined as highly virulent strain^a^**	**Literature**	**Additional information**	**GenBank ID**	**ORF1a**	**ORF1b**	**ORF2**	**ORF3**	**ORF4**	**ORF5**	**ORF6**	**ORF7**
AUT15-33	1	2015	Yes	(16, 26)	Austria	MT000052	7,188	4,374	750	798	552	606	522	387
94881	1				Vaccine strain (Reprocyc® PRRS EU)	KT988004	7,050	4,374	750	798	552	606	522	387
DV	1				Vaccine strain	MW674755	7,191	4,374	750	798	552	606	522	387
96V198	1				Vaccine strain	MK876228	7,191	4,374	750	792	546	606	522	387
Amervac	1				Vaccine strain	GU067771	7,191	4,374	750	798	552	606	522	387
Lelystad virus	1	1991		(27–29)	The Netherlands	NC_043487	7,191	4,374	750	798	552	606	522	387
Olot/91	1	1991		(30, 31)	Spain	KF203132	7,191	4,374	750	798	552	606	522	387
07V063	1	2007		(15, 32)	Belgium	GU737264	7,107	4,374	750	798	552	606	522	387
GER09-613	1	2009		(33)	Germany	KT344816	7,188	4,374	750	798	552	606	522	387
9625/2012	1	2012	Yes^b^	(14)	Hungary	KJ415276	7,191	4,374	750	798	552	606	522	387
13V091	1	2013	Yes	(15)	Belgium	KT159248	7,116	4,374	750	795	549	606	522	387
13V117	1	2013		(15)	Belgium	KT159249	7,107	4,374	750	798	552	606	522	387
AUT13-883	1	2013		(33)	Austria	KT326148	7,188	4,374	750	798	552	606	522	387
AUT14-440	1	2014		(33)	Austria	KT334375	7,152	4,374	750	762	516	606	522	387
PR40_2014	1	2014	Yes	(6, 34)	Italy	MF346695	6,732	4,374	750	792	546	606	522	387
WestSib	2	2013	Yes	(28)	Russia	KX668221	7,134	4,364	750	720	555	651	522	387
Lena	3	2007	Yes	(13, 35)	Belarus	JF802085	7,101	4,383	750	750	555	606	522	387
Tyu16	Non-subtypeable^c^	2016		(5, 36)	Russia	MT008024	7,035	4,368	750	711	549	606	522	378
VR2232	Outgroup PRRSV-2	1992	Yes	(37–39)	USA	EF536003	7,512	4,370	771	765	537	603	525	372

a *According to the corresponding literature.*

b *Assessment of virulence of this strain based on field observations. In contrast to the other strains, no experimental infection trial was conducted (14).*

c *According to the authors of the cited publication, Tyu16 belongs to PRRSV-1 with considerable distance to other PRRSV-1 strains (36). In ORF7, this isolate forms a monophyletic group with subtype 1 strains, whereas in ORF5 there is a grouping with subtype 2 strains. In literature, these strains were described as non-subtypeable (5)*.

### Experimental Procedures

After 1 week of acclimation in the BSL-2 facility, NV–I gilts (*n* = 4) and V–I gilts (*n* = 4) were inoculated with PRRSV-1 isolate AUT15-33 on gestation day 84, both intramuscularly and intranasally as described above. The day of inoculation was considered as day 0 post-infection (0 dpi). The control gilts [NV–NI gilts (*n* = 4) and V–NI gilts (*n* = 4)] were similarly mock-infected with Dulbecco's modified Eagle's medium. A clinical examination of individual animals was performed daily throughout the entire study period, including assessment of behavior, appetite, dyspnea, cough, nasal discharge, and measurement of rectal temperature. Additionally, the gilts were weighed on the day of inoculation and at 17 dpi, and the average daily weight gain (ADG) was calculated. Blood samples were taken by puncture of the jugular vein on the day of the first (−142 dpi) and the third (−31 dpi) vaccination and at 0, 6, 14, and 17 dpi. Blood from the intracardiac puncture was also collected at necropsy. PRRSV reverse transcription quantitative polymerase chain reaction (RT-qPCR) analyses were performed from the serum collected at 0, 6, 14, and 17 dpi and at necropsy. ELISA (IDEXX PRRS X3 Ab Test®) analyses were performed in serum samples from all gilts on the day of the first vaccination, at 0, 6, and 14 dpi, and at necropsy. In addition, V–NI and V–I gilts were re-investigated on the day of the third vaccination.

At 21 ± 2 dpi, an intravenous injection of ketamine (Narketan® 100 mg/ml, Vetoquinol Österreich GmbH, Vienna, Austria; 10 mg/kg body weight) and azaperone (Stresnil® 40 mg/ml, Elanco GmbH, Cuxhaven, Germany; 1.5 mg/kg body weight) was applied before the gilts were euthanized by an intracardiac injection of T61® (embutramide, mebezonium iodide, and tetracaine hydrochloride; Intervet GesmbH, Vienna, Austria; 1 ml/10 kg body weight). Necropsy was performed, including the reproductive tracts of the gilts and each of the fetuses. Tissue samples were collected from the lungs, tonsils, tracheobronchial lymph nodes, and uterine lymph nodes of each gilt for viral load quantification.

After the uterus was removed, it was placed in a trough, rinsed with tap water in order to remove the maternal blood, and opened at the anti-mesometrial side starting at the tip of each horn. The fetuses were numbered according to their location in the uterus, with the ones closest to the ovary being “L1” in the left horn and “R1” in the right horn. Fetal weight and crown–rump length (CRL) were assessed, and the fetal preservation status was evaluated and classified according to Ladinig et al. (41) as viable (VIA), meconium-stained (MEC), decomposed (DEC), autolyzed (AUT), or mummified. The mummified fetuses were excluded from further analysis. The umbilical cords were clamped close to the fetal belly, and serum samples were collected from each fetus in good preservation (VIA or MEC) directly from the heart. The fetuses and the corresponding uterine segments were removed and placed on separate trays in order to prevent cross-contamination. The fetuses were dissected, and the cervical and thoracic parts of the thymus were collected. The combined fetal placenta and endometrium were trimmed off the myometrium and manually separated from each other. Pieces of maternal endometrium and fetal placenta were collected for RT-qPCR and stored at −80°C until further processing.

### PRRSV RT-qPCR

Sampled sera were extracted with the cador Pathogen Kit for viral nucleic acid purification in a QiaCubeHT instrument (QIAGEN GmbH, Hilden, Germany) according to the manufacturer's protocol. To avoid clogging of the channels of the extraction plates, tissue and organ sections (50 mg) were homogenized in 600 μl Qiazol (QIAGEN) using 3 stainless steel beads (3 mm) in a 2-ml screw-capped tube (SARSTEDT AG & Co., KG, Germany) at full speed in a TissueLyser II instrument (QIAGEN) for 3 min. The homogenate was briefly centrifuged, and 300 μl of chloroform was added. The capped tubes were thoroughly vortexed and centrifuged for phase separation at 13, 000 × *g* for 5 min. Two hundred microliters of the aqueous phase was collected and further processed using the cador Pathogen Kit in a QiaCubeHT instrument (QIAGEN) according to the manufacturer's protocol.

Two microliters of the eluted RNA was used for ORF7-specific RT-qPCR using the Luna Onestep RT PCR Kit (New England Biolabs). The primer sequences were adapted from Egli et al. (42) to fit the sequence of PRRSV-1 strain AUT15-33 (PRSq1 forward: TCAACTGTGCCAGTTGCTGG, PRSq2 reverse: TGRGGCTTCTCAGGCTTTTC, and PRSq3 probe: 5′Fam-CCCAGCGYCRRCARCCTAGGG Tamra-3′). For RT-qPCR of the vaccine strain contained in ReproCyc® PRRS EU, the primer set PRSq1 forward: TCAACTGTGCCAGTTGCTGG, PRSq4 reverse: TGTGGCTTCTCAGGCTTCTTC, and PRSq5 probe: 5′Fam-CCCAGCGCCAGCAAYCTAGGG Tamra-3′ were employed.

Previous studies suggest that cell-free blood contains larger amounts of exosomes, which also provide β-actin (43, 44). As extraction control, porcine β-actin was quantified by qPCR in all samples. The detection of β-actin was based on a previously described protocol by Toussaint et al. (45). Single runs were performed for both PRRSV and β-actin PCR with a cut-off value set at Cycle threshold (Ct) 38 since, for the PRRSV-specific PCR, this was the limit to reproducibly and reliably detect 100 copies.

The absolute quantity of the genome equivalents (GE) was calculated from serially diluted SP6 transcripts of cloned AUT15-33 cDNA fragment 13261−3′ end in a pGEM-T (Promega GmbH, Germany) plasmid (pLS69). Transcripts were generated with 0.5 μg of AclI linearized pLS69 plasmid DNA using 20 units of SP6 polymerase (New England Biolabs) in a 20-μl reaction. Template DNA was digested with DNAse I (New England Biolabs), and the RNA was purified using the RNeasy Kit (QIAGEN). The RNA concentration was determined with a Quantus fluorometer and a RNA-specific fluorescent dye (Promega). The number of genome molecules was calculated by multiplication of the RNA concentration with Avogadro's number divided by the molecular mass of an AUT15-33-specific SP6 transcript. Quantitative PCR was done in an Applied Biosystem 7300 instrument (Applied Biosystems, Thermo Fisher Scientific Inc., USA).

### Statistical Analysis

Clinical observations of the gilts and serological responses were investigated descriptively. Variables without repeated measures were modeled using linear models (LM). In the case of repeated measures for fetuses or gilts, the individual fetus or gilt was used as a random effect in a mixed-effects linear model. The following model assumptions were always checked: (1) the normality of residuals was checked by the Shapiro–Wilk normality test, (2) the homogeneity of variances between groups was checked with Bartlett test, and (3) the heteroscedasticity (constancy of error variance) was checked with Breusch–Pagan test. In case the assumptions were not satisfied, robust linear or robust mixed-effects linear models were applied.

Thus, group influences on average daily weight gain and differences between treatment groups in the percentage of viable fetuses per litter were studied using an LM. After calculating the area under the curve (AUC) of gilt serum samples according to Ladinig et al. (46), viral loads of gilt serum AUC and gilt tissue samples were compared between V–I and NV–I gilts also by LM. The fetal weight and crown–rump length of VIA and MEC fetuses were explored *via* linear mixed-effects models. The rectal temperature of gilts from all treatment groups and the viral load in fetal compartments (serum, thymus, endometrium, and placenta) of fetuses from V–I and NV–I gilts were studied by means of robust mixed-effects linear models. Contrasts (odds ratios) between treatment groups with regard to fetal preservation status were calculated by the generalized linear mixed-effects logistic regression after dichotomizing the fetal preservation in 2 categories: viable and non-viable (including the categories MEC, DEC, and AUT).

Moreover, the influence of 5 predictors (gilt serum AUC and gilt tissue samples of the tonsil, lung, tracheobronchial lymph node, and uterine lymph node) on the proportion of PCR-positive fetuses (serum and/or thymus) as response variable was first checked univariately as described by Dohoo et al. (47). The variables which showed a *p*-value <0.2 in the univariate analyses then became part of the multiple linear regression. All contrasts (differences) between particular groups were assessed after model-fitting by the estimated marginal means (emmeans) with no *p*-value correction for multiple comparisons due to the small sample sizes and the rather explorative nature of our study. The results with a *p*-value <0.05 were considered statistically significant, while the results with a *p*-value <0.1 were considered suggestive. All models were conducted using R Statistical language [version 4.0.3; (48)]. Fetal preservation and PRRSV-1 AUT15-33-specific RT-qPCR results in fetal samples were displayed using Microsoft Excel (Office 2016, Microsoft, Redmond, WA, USA). The course of rectal temperature and serological responses was visualized using GraphPad Prism 9.0.0 for Windows (GraphPad Software, San Diego, CA, USA). Figures of predicted gilt ADG values, predicted values for the percentage of VIA fetuses per litter, and viral load in gilt tissues were produced in R.

## Results

### AUT15-33 Full-Length Sequence

The AUT15-33 genome consists of 15,093 nucleotides (polyA not included). The size of the open reading frames (ORFs) compared to the other selected viruses is compared in Table 1. The only difference to LV is the single deletion of one codon in the variable NSP2 region (corresponding to Val672 of LV). On the basis of the full-length sequence, no immediate relatives are apparent. BLAST calculates the best match (87.9%) with LV indicating a considerable distance. Recently published ORF5 sequences from Hungary [7485_NEBIH_2016_HU [Acc. no. MN102319], 55548_NEBIH_2016_HU [MN102281], and 54292_NEBIH_2014_HU [MN102275]] were 98.5, 97.7, and 95.5% homologous, respectively (49). There is a similar match with two Slovenian isolates [230A/2018 [MK814109] and 212A/2017 [MK814097]] with 98.4% and 98.0% homology. All other isolates were <95% homologous in ORF5. For ORF7, a 95.4% homology was detected with the Croatian strain CRO_PRRSV_3 (KF498723) (50). Phylogenetic tree analyses of the described whole-genome sequences (Figure 2A) revealed a closest relationship to the Belgian isolate 13V091 (15). Within the vaccine strains, a closer relationship was shown to 94881, which is the strain of ReproCyc® PRRS EU. Phylogenetic tree analyses of the described ORF5 sequences are displayed in Figure 2B.

### Clinical Signs and Serological Responses

All gilts were clinically healthy upon arrival. Overall, only mild clinical signs were recorded after challenge (V–I and NV–I gilts). The most frequent clinical presentation was mild nasal discharge, which was recorded in both vaccinated and non-vaccinated inoculated animals. Since the signs were mild and only observed on single days, no further statistical analysis was conducted. Some gilts developed lameness during the course of the experiment. One NV–I gilt (gilt 22), the most severely affected, developed a phlegmon at the right rear foot and was treated with antimicrobial and non-steroidal anti-inflammatory drugs according to the manufacturer's instructions (metamizole sodium: Novasul® 500 mg/ml, Richter Pharma AG, Austria, and enrofloxacin: Baytril® RSI 100 mg/ml, Bayer AG, Austria). In addition, mild lameness was observed in one NV–NI gilt (gilt 2) starting on 5 dpi and remained until the end of the experiment. Both animals were subsequently excluded from the analysis of average daily gain, rectal temperature, fetal weight and CRL.

**Figure 3:**
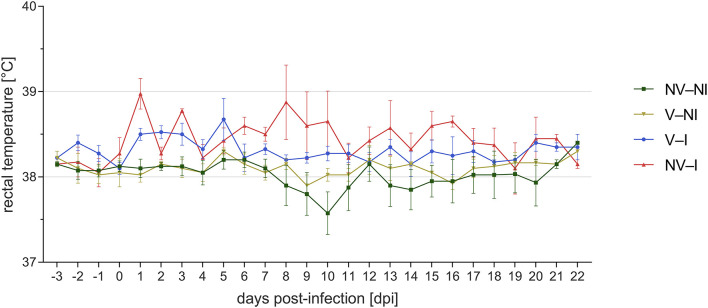
Course of mean rectal temperature and standard error of gilts from the four treatment groups after porcine reproductive and respiratory syndrome virus infection per day post-infection. Gilts 2 and 22 were excluded from visualization. NV–NI, non-vaccinated and non-infected gilts; V–NI, vaccinated and non-infected gilts; V–I, vaccinated and infected gilts; NV–I, non-vaccinated and infected gilts.

**Figure 4:**
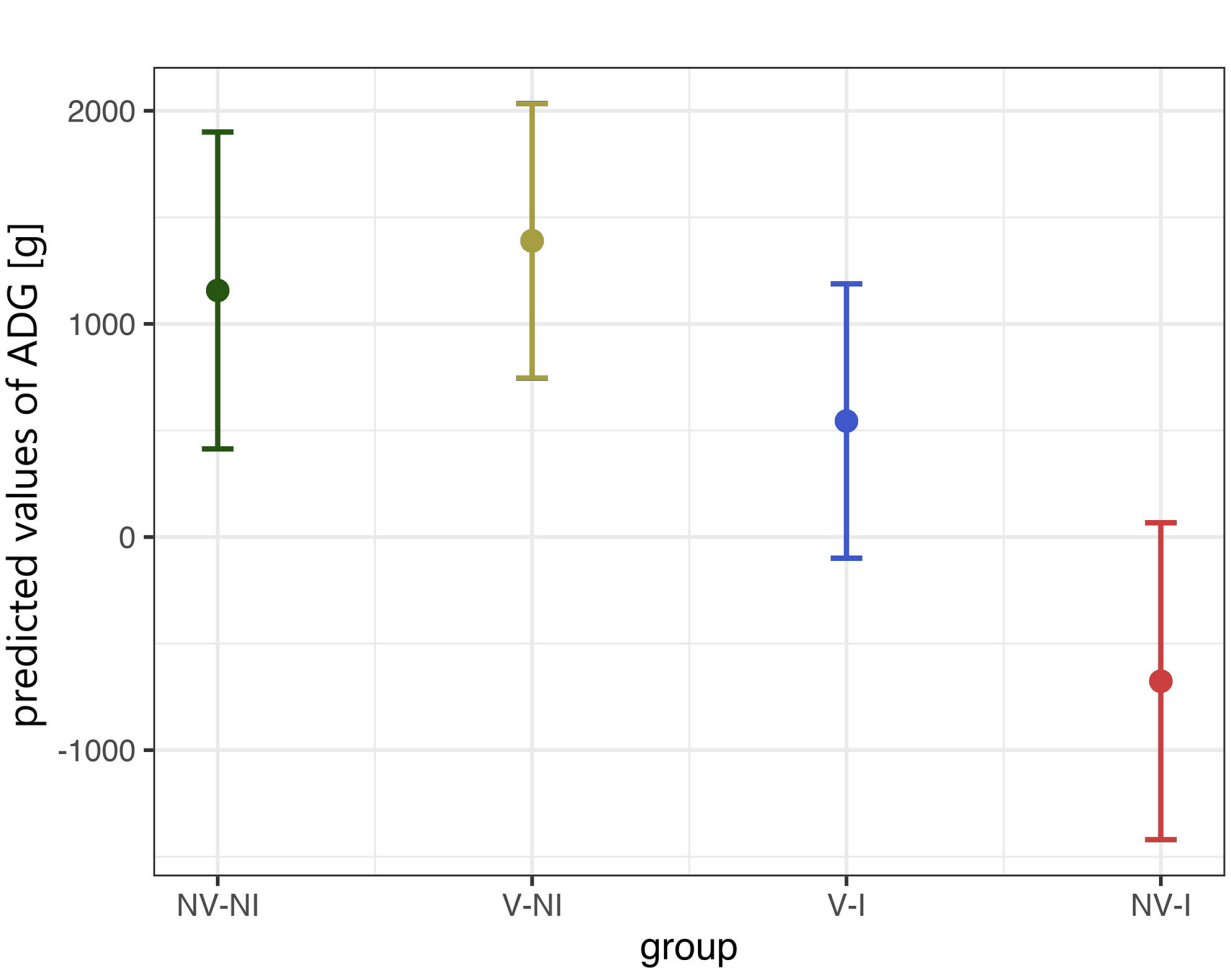
Predicted values of average daily weight gain (ADG) from the day of challenge up to 17 days post-infection in relation to treatment group, calculated with a linear model. Gilts 2 and 22 were excluded from this analysis. The values in the Y-axis represent the predicted daily weight gain in gram. The data points indicate the estimated marginal means of each group, and the error bars illustrate the respective confidence interval (confidence level of 95%). NV–NI, non-vaccinated and non-infected gilts; V–NI, vaccinated and non-infected gilts; V–I, vaccinated and infected gilts; NV–I, non-vaccinated and infected gilts.

Rectal temperature was significantly higher in NV–I and V–I gilts compared to NV–NI and V–NI gilts. Significant differences were found between NV–NI and V–I gilts (*p* = 0.034), NV–NI and NV–I gilts (*p* < 0.001), V–NI and V–I gilts (*p* = 0.001), and V–NI and NV–I gilts (*p* < 0.001). Additionally, rectal temperatures were numerically higher in NV–I gilts compared to V–I gilts (*p* = 0.137). The course of the rectal temperature in the four treatment groups is displayed in Figure 3. In total, loss of body weight was observed in five gilts during the days post-infection: one NV–NI gilt (gilt 2), which showed locomotory disorders, one V–I gilt (gilt 16), which did not show any obvious clinical signs but mildly increased rectal temperature for 1 day (39.1 degrees), and three NV–I gilts (gilts 22, 23, 24), including the gilt with locomotory problems. The average daily weight gain of NV–I gilts was significantly lower compared to NV–NI gilts (*p* = 0.007) and V–NI gilts (*p* = 0.002). Significant differences were also found between V–I and NV–I gilts (*p* = 0.035). Predicted values of ADG from the day of challenge up to 17 days post-infection in relation to treatment group are shown in Figure 4.

**Figure 5:**
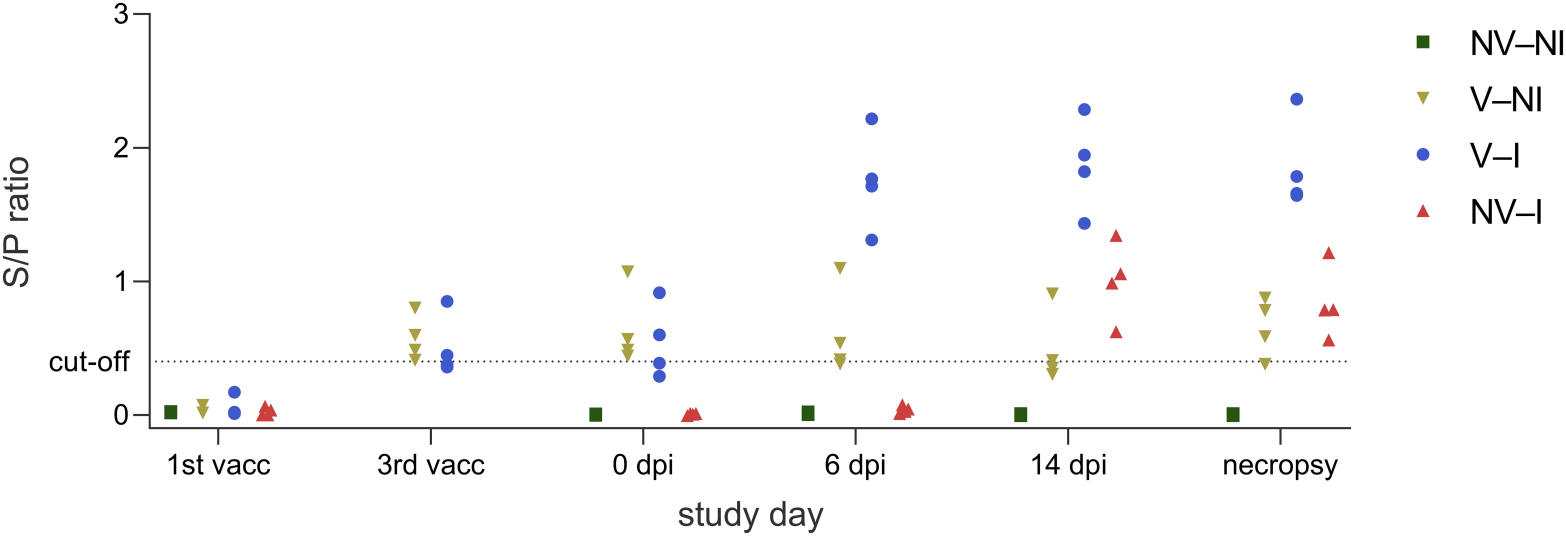
Serological responses of gilts from the four treatment groups (NV–NI, non-vaccinated and non-infected; V–NI, vaccinated and non-infected; V–I, vaccinated and infected; NV–I, non-vaccinated and infected) at the timepoint of the first vaccination, third vaccination, 0 dpi, 6 dpi, 14 dpi, and on the day of necropsy, presented as an aligned dot plot. In contrast to the other timepoints, only V–NI and V–I gilts were examined on the day of the third vaccination. The ELISA was considered positive at an S/P ratio of ≥0.4.

All gilts were negative for antibodies against PRRSV-1 nucleoprotein at the first vaccination timepoint. V–NI and V–I gilts were re-tested on the day of third vaccination. At this timepoint, all vaccinated gilts had S/P ratios above the cut-off or close to the cut-off value of the ELISA kit (S/P ratio of ≥0.4). On the day of PRRSV infection, NV–NI and NV–I gilts were negative for anti-PRRSV antibodies while V–NI and V–I gilts were positive with the exception of two gilts which had S/P ratios slightly below the cut-off on the day of the third vaccination (gilts 15 and 16). After inoculation, the anti-PRRSV antibody response was detected faster in V–I gilts compared to NV–I gilts. All V–I gilts showed a clearly positive result in the ELISA at 6 dpi while all NV–I gilts were still negative 6 days after infection. By 14 dpi, all NV–I and V–I gilts had S/*P*-values above the propagated cut-off value. Serological responses per treatment group are visualized in Figure 5.

### Fetal Preservation Status

**Figure 6:**
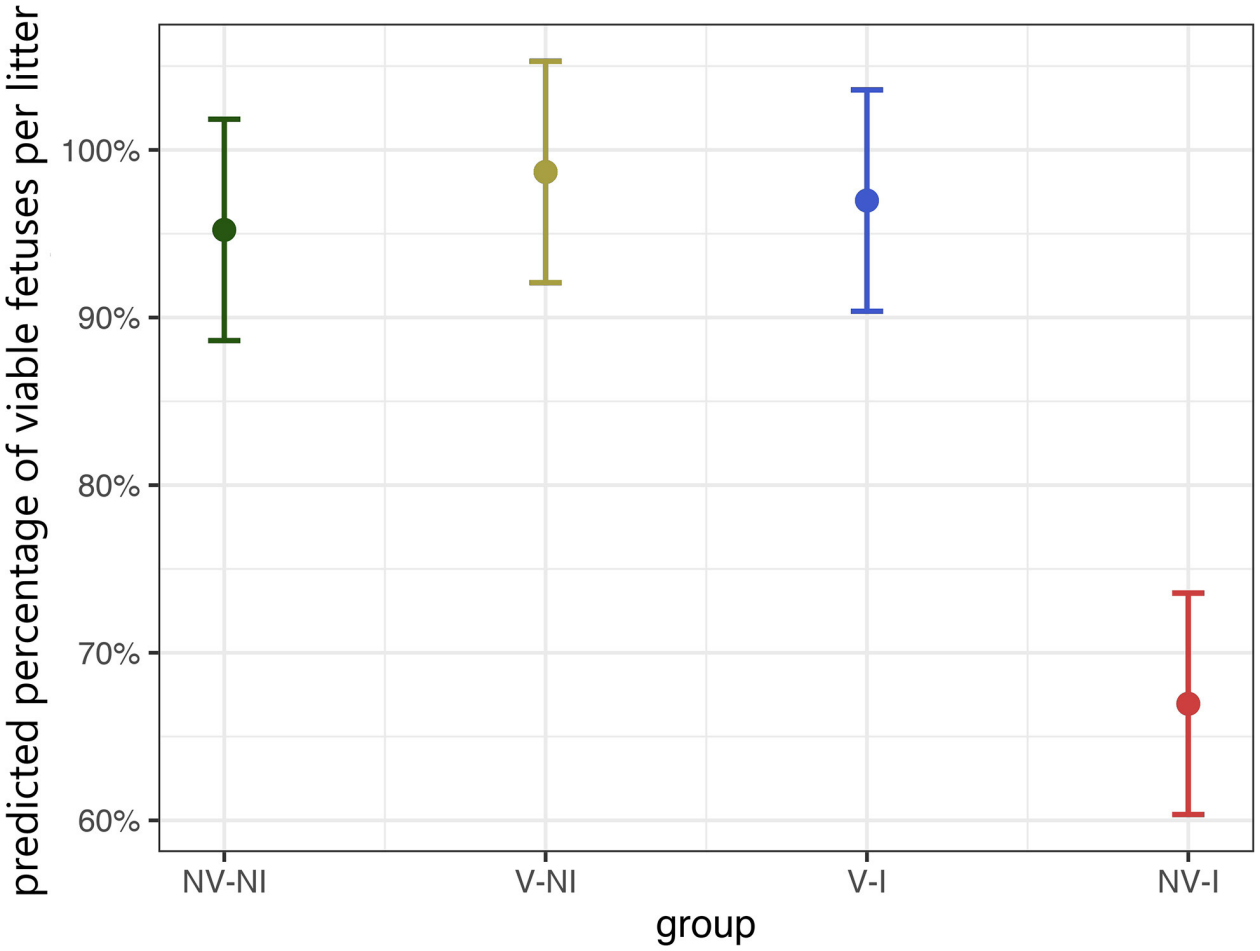
Predicted values of the percentage of viable (VIA) fetuses per litter in relation to treatment group, calculated with a generalized linear mixed-effects logistic regression model. Fetal preservation status was dichotomized into two categories: VIA and non-VIA. The category VIA includes fetuses with physiologically white to purple skin color without meconium accumulation and physiological pulsing and bluish shimmering umbilical cord without visible edematous areas. The non-VIA category includes meconium-stained, decomposed, and autolyzed fetuses. The data points indicate the estimated marginal means of each group, and the error bars illustrate the respective confidence interval (confidence level of 95%). NV–NI, non-vaccinated and non-infected gilts; V–NI, vaccinated and non-infected gilts; V–I, vaccinated and infected gilts; NV–I, non-vaccinated and infected gilts.

All fetuses from NV–NI and V–NI gilts were categorized as VIA or MEC. No dead fetuses were found in those gilts and the percentage of MEC fetuses was low (3.6% and 1.5%). In V–I gilts individual fetuses were MEC (3.4%) or DEC (1.7%); however, the highest percentage of fetuses from this treatment group was categorized as VIA (94.9%). An increased number of mummified fetuses, which would indicate the presence of other infectious agents, could not be detected. Most severe deviations in fetal preservation status were observed in NV–I gilts. The absolute percentage of non-viable fetuses in gilts from this treatment group was 44.1%, with 22.1% of fetuses being categorized as MEC, 7.3% as DEC and 14.7% as AUT. Only 55.9% of fetuses did not show external changes and were considered as VIA. Predicted values of the percentage of VIA fetuses per litter in relation to treatment group are displayed in Figure 6. Comparing the different treatment groups, significant differences in the percentage of viable fetuses per litter were found between NV–NI and NV–I gilts (*p* < 0.001), V–NI and NV–I gilts (*p* < 0.001), as well as V–I and NV–I gilts (*p* < 0.001), but not between other treatment groups.

**Figure 7:**
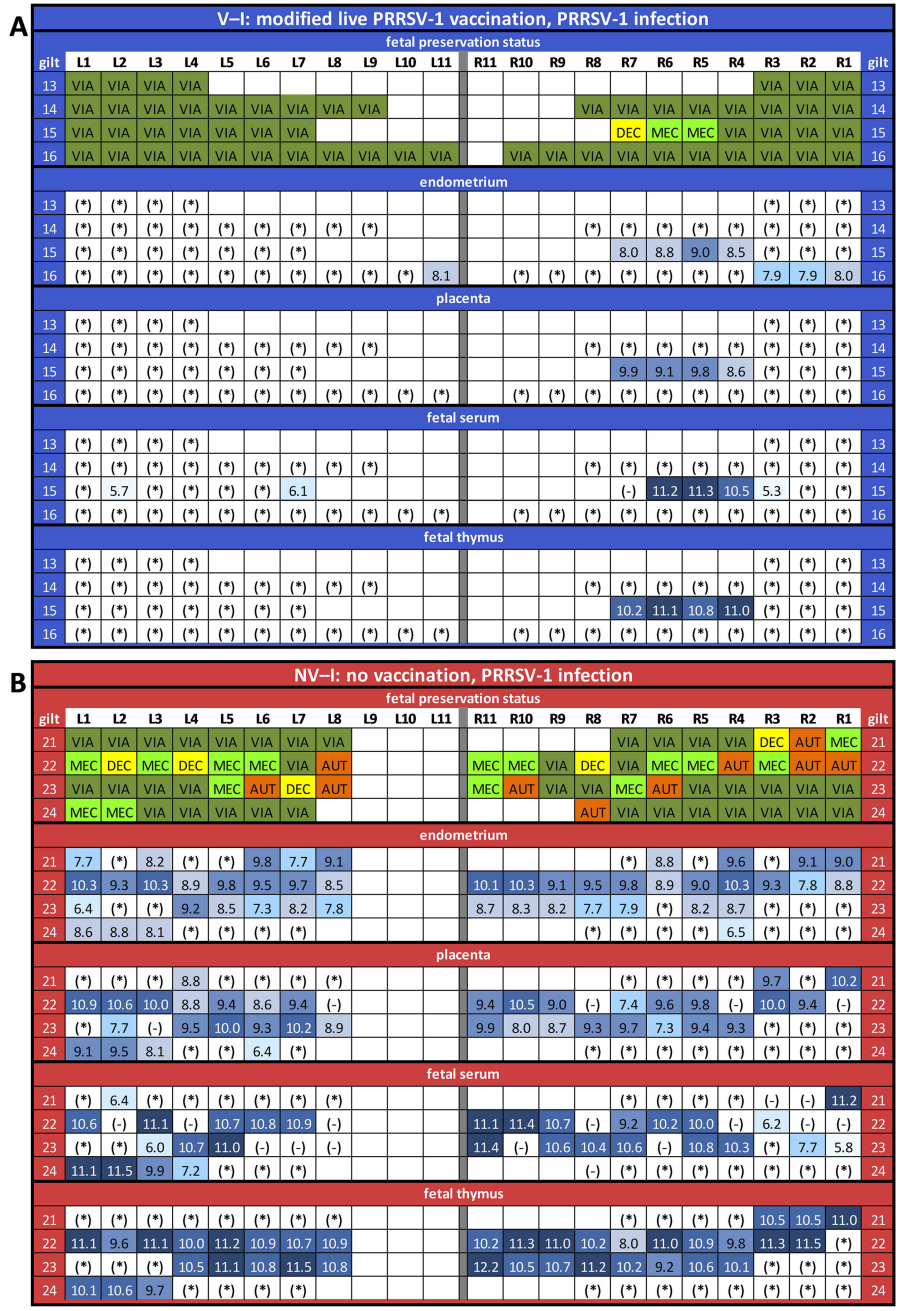
Fetal preservation and porcine reproductive and respiratory syndrome virus-1 AUT15-33-specific RT-qPCR results in fetal samples collected at necropsy (presented as log_10_-transformed genome equivalents per gram of tissue or milliliter of serum) for the vaccinated and infected (V–I) gilts **(A)** and for the non-vaccinated and infected (NV–I) gilts **(B)**. The fetuses were numbered according to their location in the uterus, with the ones closest to the ovary being “L1” in the left horn and “R1” in the right horn. Each line represents one litter. For the fetal preservation, colors represent the different categories (viable in dark green, meconium-stained in light green, decomposed in yellow, and autolyzed in orange). For the RT-qPCR results, colors represent the number of genome equivalents per gram of tissue or milliliter of serum (light blue to dark blue). *, below detection limit; -, not sampled. The RT-qPCR results are presented for the endometrium, fetal placenta, fetal serum, and fetal thymus of each piglet.

Fetuses from NV–NI, V–NI and V–I gilts had a significantly higher odds of being viable compared to fetuses from NV–I gilts [NV–NI vs. NV–I: OR = 26.77, 95% CI = (2.69, 266.77), *p* = 0.005; V–NI vs. NV–I: OR = 79.23, 95% CI = (4.99, 1258.92), *p* = 0.002; V–I vs. NV–I: OR = 23.31, 95% CI = (2.32, 233.99), *p* = 0.007]. Results of the fetal preservation categories by treatment groups are presented in Figures 7A,B. No significant differences were detected in fetal weight of VIA and MEC fetuses [NV–NI: 762 (±46.7) g; V–NI: 833 (±38.1) g; V–I: 787 (±39.6) g; NV–I: 739 (±45.4) g] and CRL [NV–NI: 23.0 (±0.51) cm; V–NI: 23.2 (±0.41) cm; V–I: 23.0 (±0.43) cm; NV–I: 22.3 (±0.49) cm] between the four treatment groups. The given values are the emmeans of the group with the standard error in square brackets.

### RT-qPCR Results of Gilt Serum and Tissue Samples

No PRRSV RNA was detected in any tissue or serum samples from NV–NI and V–NI gilts by the AUT15-33-specific RT-qPCR. Therefore, only NV–I and V–I gilts were compared in the performed analyses. Furthermore, no PRRSV RNA was detected in serum samples of inoculated animals prior to challenge on 0 dpi using the RT-qPCR specific for the ReproCyc® PRRS EU vaccine. After challenge, the serum viral load AUC was significantly lower in V–I gilts compared to NV–I gilts (*p* < 0.001) using the AUT15-33-specific RT-qPCR. Apart from two V–I gilts, which were viremic at 6 dpi, no viremia was detected in serum of vaccinated gilts after PRRSV challenge (Table 2). In contrast, all NV–I gilts were viremic from 6 dpi until 17 dpi. Additionally, two NV–I gilts were positive by AUT15-33-specific RT-qPCR in serum on the day of necropsy (Table 2).

**Table 2:** PRRSV-1 AUT15-33 detection in serum at certain days post-infection (dpi) and tissue samples from vaccinated–infected gilts (V–I) and non-vaccinated-infected gilts (NV–I) collected at necropsy on 21 ± 2 dpi.

**Gilt**	**Group**	**0 dpi**	**6 dpi^a^**	**14 dpi^a^**	**17 dpi^a^**	**21 ± 2 dpi^a^**	**Serum area under the curve^b^**	**Lung^c^**	**Tonsil^c^**	**Tracheobr.ln.^c^**	**Uterineln.^c^**
13	V–I	(*)	(*)	(*)	(*)	(*)	0.00	(*)	8.47	10.21	8.60
14	V–I	(*)	5.12	(*)	(*)	(*)	35.81	10.56	8.98	7.45	8.77
15	V–I	(*)	5.97	(*)	(*)	(*)	41.78	(*)	9.36	8.77	8.97
16	V–I	(*)	(*)	(*)	(*)	(*)	0.00	(*)	9.20	7.90	8.86
21	NV–I	(*)	8.66	6.07	7.05	(*)	118.70	6.89	9.81	10.20	10.33
22	NV–I	(*)	9.78	8.67	7.88	7.18	158.11	8.36	10.74	10.94	10.78
23	NV–I	(*)	9.24	6.11	6.42	(*)	120.72	(*)	9.80	8.95	10.38
24	NV–I	(*)	9.62	7.52	6.47	6.27	143.91	9.25	9.15	10.00	9.96

*The asterisk indicates below detection limit.*

a *Log_10_-transformed genome equivalents/ml serum.*

b *The value for serum area under the curve was calculated in accordance with Lading et al. (46).*

c *Log_10_-transformed genome equivalents/g tissue (lung, tonsil, tracheobronchial and uterine lymph node)*.

**Figure 8:**
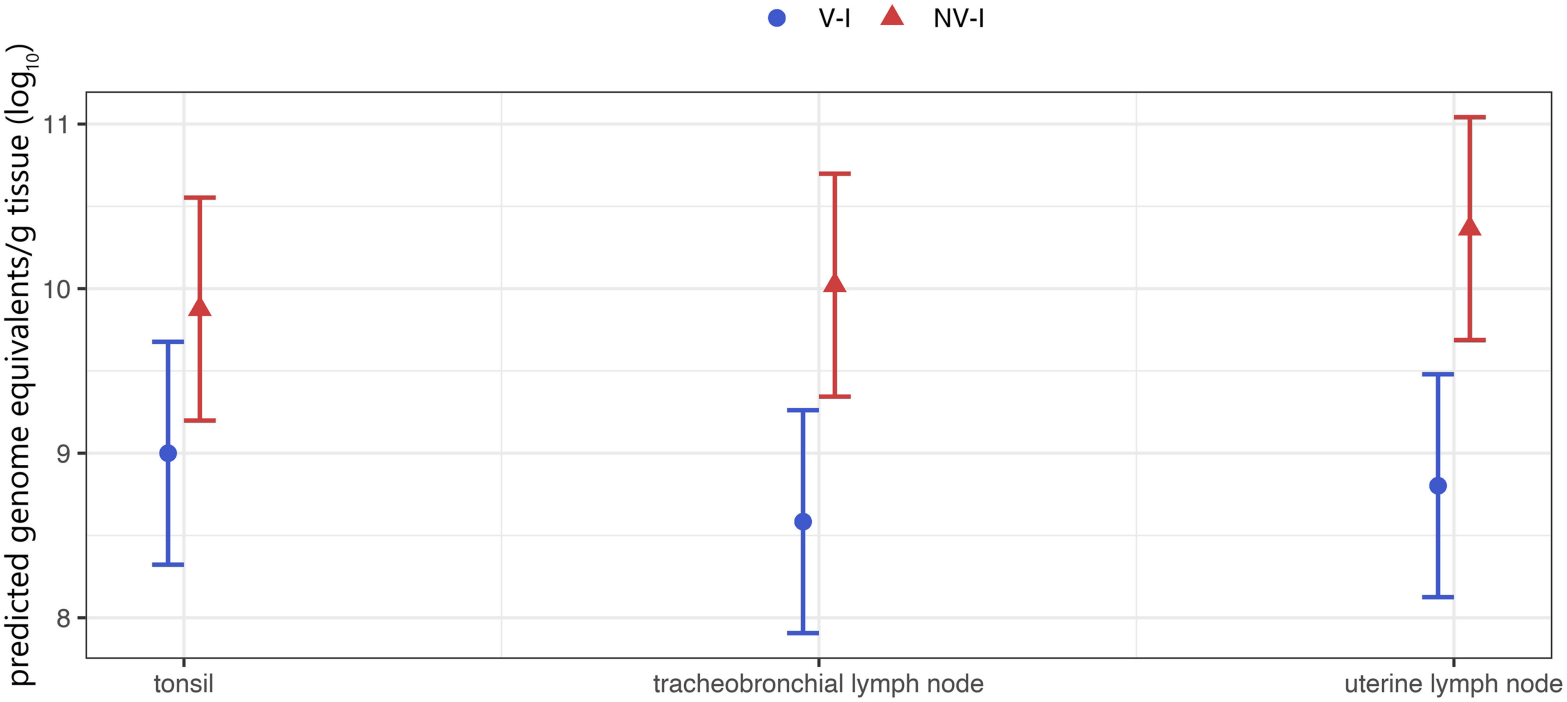
Tissue viral load of the gilts (tonsil, tracheobronchial and uterine lymph node) per treatment group. The porcine reproductive and respiratory syndrome virus (PRRSV) RNA was below the detection limit of PRRSV-1 AUT15-33-specific RT-qPCR in all tissue samples from the control animals (NV–NI and V–NI gilts). Therefore, only the two infected groups are shown. V–I, vaccinated and infected gilts, blue color; NV–I, non-vaccinated and infected gilts, red color. The values in the Y-axis represent viral load as determined by RT-qPCR. The data points indicate the estimated marginal means of each group, and the error bars illustrate the respective confidence interval (confidence level of 95%).

Investigated tonsils, tracheobronchial and uterine lymph node samples of all inoculated gilts were RT-qPCR positive at time of necropsy. The lowest detection rate and the lowest viral load were found in the lungs. Due to the low viral detection rate in lung tissue of V–I gilts, a comparison between the two infected groups was not possible. The viral load in the tracheobronchial lymph nodes and the uterine lymph nodes was significantly lower in V–I gilts compared to NV–I gilts (*p* = 0.009 and *p* = 0.005, respectively). Additionally, the viral load in the tonsils was numerically lower in V–I gilts compared to NV–I gilts (*p* = 0.090). Tissue viral load of tonsils, tracheobronchial and uterine lymph nodes for V–I and NV–I gilts are visualized in Figure 8.

The proportion of PRRSV positive fetuses (serum and/or thymus) was associated with several properties of the gilts. If the serum viral load AUC of the gilts rose by one unit, the proportion of PRRSV positive fetuses per litter rose by 0.4 percentage points (*p* = 0.044). Additionally, if the viral load of the uterine lymph node rose by one log_10_ GE/g tissue, the proportion of PRRSV positive fetuses rose by 34 percentage points (*p* = 0.021). If the viral load of the tonsils rose by one log_10_ GE/g tissue, the proportion of PRRSV positive fetuses rose by 47 percentage points (*p* = 0.009). Viral load of the lung and tracheobronchial lymph nodes was not significantly associated with the proportion of PRRSV positive fetuses per litter. Raw data of serum and tissue viral load of the gilts and fetuses (Ct-values and genome equivalents) as well as results of the β-actin-PCR (Ct-values) are presented in Supplementary Table S2.

### RT-qPCR Results of the Fetal Compartment

All samples from control litters (NV–NI and V–NI gilts) yielded negative results by AUT15-33-specific RT-qPCR, including the fetuses from the control groups that were MEC (NV–NI gilts: *n* = 2; V–NI gilts: *n* = 1). Serum could only be collected from VIA and MEC fetuses. The viral load of fetuses from V–I and NV–I gilts could be quantified in all DEC (*n* = 6) and MEC fetuses (*n* = 17), in 80% of AUT fetuses (*n* = 8), and in 19.2% of VIA fetuses (*n* = 18) in either the serum or thymus sample. Viral load in fetal serum according to fetal preservation status was on average 1.64 [±0.36] log_10_ GE/ml in VIA compared to 10.67 [±0.30] log_10_ GE/ml in MEC fetuses. Viral load in thymus was 1.10 [±0.33] log_10_ GE/g in VIA, 10.95 [±0.12] log_10_ GE/g in MEC, 10.34 [±0.27] log_10_ GE/g in DEC and 8.39 [±1.41] log_10_ GE/g in AUT fetuses.

Comparing fetal serum RT-qPCR results of V–I and NV–I gilts, fetuses from NV–I gilts [5.28 (±2.50) log_10_ copies/ml] had a numerically higher viral load than fetuses of V–I gilts [0.14 (±2.50) log_10_ copies/ml, *p* = 0.146]. Comparing the viral load in fetal thymus of VIA and MEC fetuses between these two groups, the viral load in fetuses from NV–I gilts [5.23 (±1.74) log_10_ copies/ml] was higher compared to fetuses from V–I gilts [0.47 (±1.76) log_10_ copies/ml, *p* = 0.054]. In V–I gilts, PRRSV was only detected in fetal serum and fetal tissues (placenta and/or thymus) of one litter (gilt 15; 50% of fetuses affected) (Figure 7A), which was the only one V–I gilt with fetuses showing gross pathologic changes at necropsy.

For maternal endometrium, viral load was significantly lower in fetuses from V–I gilts [0.68 (±1.22) log_10_ copies/ml] compared to fetuses from NV–I gilts [6.00 (±1.20) log_10_ copies/ml; *p* = 0.002]. Significant differences were also present in the viral load in the fetal placenta between fetuses from V–I gilts [0.39 (±1.60) log_10_ copies/ml) and fetuses from NV–I gilts [4.91 (±1.59) log_10_ copies/ml; *p* = 0.045]. Viral load in fetal thymus was positively associated with the viral load in both endometrium and placenta (*p* < 0.001). In total, 14 out of 127 fetuses (four fetuses from V–I gilts, 10 fetuses from NV–I gilts) from infected gilts were RT-qPCR negative in both thymus and serum, despite the detection of virus in the respective endometrial sample. With the exception of one, all fetuses were VIA. Fetal preservation status and PRRSV-1 AUT15-33-specific RT-qPCR results in fetal samples collected at necropsy for V–I and NV–I gilts are displayed in Figures 7A,B, respectively.

## Discussion

Massive reproductive losses during fast spreading local outbreaks in Lower Austria in spring 2015 suggested that the novel AUT15-33 PRRSV isolate is of considerable virulence (16). Since its isolation, AUT15-33 has been causing a rather large epidemic in central Europe. Even 6 years after its first detection, we observe AUT15-33 derivatives causing significant health problems in pig farms including respiratory pathologies and reproductive failure. Phylogenetic analyses and homology calculations of AUT15-33 with full ORF2-7, ORF5 and ORF7 sequences were already carried out in a previous publication (16). Since then, several ORF5 sequences with close similarity to AUT15-33 have been revealed in different countries. Close nucleotide homologies (≥95%) with Hungarian and Slovenian field strains were detected; unfortunately, no further information on the virulence of these strains is available (49). For the Croatian strains which still show the highest homology in ORF7, only these ORF7 sequences are published, thus no larger sequence comparison was possible (16). This evidence supports a regional cluster of AUT15-33 in the south-eastern part of Central Europe. AUT15-33 shows considerable distance (87.9%) to LV, the reference strain for PRRSV-1 subtype 1 isolated 30 years ago, whereas the gene sizes of the individual open reading frames have barely changed over time. For the phylogenetic tree, PRRSV-1 subtype 1 strains were selected, for which both the phenotype and the whole-genome sequence have been published. Phylogenetically, AUT15-33 is most closely related to the highly virulent 13V091 isolate from Belgium, which is in agreement with the phylogenetic analysis based on ORF5 nucleotide sequences (16). Among the selected attenuated strains, the vaccine strain Reprocyc® PRRS EU used in this study is phylogenetically closely related to AUT15-33 than other vaccine strains. However, both strains can be clearly characterized as heterologous strains, especially since 13V091 and the vaccine strain show only 85.3 and 85.6% total nucleotide identity compared to AUT15-33, respectively.

Severe clinical outbreaks caused by highly virulent PRRSV-2 isolates have been reported, starting in 2006, in Southeastern Asia (12). It is claimed that the presence of discontinuous deletions in the NSP2-coding region can be regarded as the hallmark genetic characteristic of highly virulent Asian PRRSV-2 isolates without being necessarily related to virulence (51). Other authors suggest that changes in individual amino acids in NSP9 are characteristic of highly virulent PRRSV-2 strains (52, 53). For PRRSV-1 subtype 3 strain Lena, the deletion of 29 amino acids in a variable region of NSP2 was described (35). A discontinuous amino acid deletion in the NSP2 coding region has also been described for the phenotypically highly virulent PRRSV-1 subtype 1 strain PR40/2014 (6). However, none of these characteristics could be detected in AUT15-33, with the exception of a single codon deletion. Nevertheless, there is no established genetic virulence marker for PRRSV-1, necessitating the experimental phenotypic determination of virulence. Consequently, the experimental proof of AUT15-33 being virulent in pregnant gilts represents an important finding as there are not many phenotypically and genotypically well-characterized PRRSV-1 strains of similar virulence. A more comprehensive genomic characterization of pathogenic PRRSV-1 isolates is mandatory to detect differences in the genome of low- and high-virulence strains, and further experimental studies are needed to confirm the virulence of PRRSV-1 strains.

An in-depth genetic and phenotypic characterization of emerging PRRSV isolates is also pivotal for the control and update of diagnostic methods, the monitoring of vaccine efficacy against new isolates, and also potential changes in virus pathogenicity and virulence. In general, the virulence of different PRRSV isolates was mainly evaluated for respiratory disease in young pigs (6, 13, 26, 36, 54, 55). Additionally, challenge experiments investigating the mechanism of PRRSV-induced reproductive failure were predominately performed with PRRSV-2 (41, 56–58). Detailed *in vitro* and *in vivo* information is available for the current Belgian PRRSV-1 subtype 1 field strain 07V063, both in a reproductive as well as a respiratory model (15, 32, 40). In the reproductive model, Karniychuk et al. reported clinical signs in intranasally infected sows (*n* = 3) as mild anorexia and depression on days 3 and 4 post-infection and a rise in body temperature in one sow of up to 38.9°C on the 2^nd^ day after infection. Viremia was detected in all sows at 5 days post-infection. A total of 45 fetuses were collected from these three sows, with seven fetuses (15.5%) showing gross pathologic lesions (32). However, other PRRSV studies in gilts and sows have often used different experimental setups and testing methods, which limit the comparability of these trials (19, 59–61). In order to contrast the clinical performance and fetal preservation status with data from a large-scale PRRSV-2 study (41), a comparable experimental design was chosen in the present study, whereby a lower number of animals was used based on the results of the previous study.

In this experiment, the gilts showed no obvious clinical signs after challenge, although inoculation was successful as verified by PRRSV detection in tissue samples of all exposed gilts. Despite the absence of obvious clinical signs, rectal temperature was significantly higher in inoculated gilts compared to that in control animals. In the PRRSV-2 study, clinical signs of respiratory disease or depression were also absent after infection, although a reduction in feed intake was evident, and the inoculated animals were significantly more likely to have a rectal temperature above 39.5°C (41). Additionally, the average daily weight gain from the day of inoculation up to 17 dpi was assessed in our study, resulting in a significantly lower ADG in NV–I gilts compared to those in NV–NI, V–NI, and V–I gilts. In contrast to gilts from all other treatment groups, which gained weight between 0 and 17 dpi, the NV–I gilts showed a quite substantial loss of weight after PRRSV infection. In the large-scale PRRSV-2 infection study, the fetuses of infected animals were significantly lighter compared to the fetuses of the control group (41). No significant differences in fetal weight and crown–rump length were detected in the current study. It has to be taken into account that only VIA and MEC fetuses were included in the evaluation. Therefore, the sample size was rather low and might explain the absence of significance. However, the fetuses from NV–I gilts were, on average, 93 g lighter compared to the fetuses from V–NI gilts. In the field, a reduced birth weight might lead to an increase in the number of weak piglets and suckling piglet mortality (62).

In relation to the fetal preservation status, the inoculation of pregnant gilts with the PRRSV strain AUT15-33 on gestation day 84 and termination at around 21 days later resulted in 44.1% of non-viable fetuses in the non-vaccinated group compared to 50.1% non-viable fetuses in the PRRSV-2 challenge model using similar experimental settings (41). In accordance with the PRRSV-2 experiment, the percentage of non-viable fetuses varied widely among litters, and the dead fetuses either were clustering in individual litters or appeared at solitary or random positions. The clustering of dead fetuses most likely results from inter-fetal PRRSV transmission, as previous studies have indicated that the adjacent fetus had a significant influence on the fetal outcome (46). In principle, our data are comparable to those of the large-scale PRRSV-2 study. Since there is no data in this experimental design for PRRSV-1 strains, our results provide new insights into PRRSV-1 pathogenicity and the effect of vaccination. To the authors' knowledge, there is no study that more closely determines the assessment of virulence in the experimental reproductive model as already been propagated for the respiratory model in growing pigs (63). Therefore, reproductive failure was used for the determination of phenotypic virulence, indicating a high virulence of AUT15-33.

The continuous and rapid evolution of PRRSV as well as the emergence of highly virulent strains has reinforced the discussion about the efficacy of currently available vaccines. Despite extensive research evaluating the protective effect of vaccination against the respiratory signs of PRRSV, only limited data on the control of reproductive failure by vaccination in an experimental setup exists (19, 64–66). Therefore, the efficacy of vaccination with a commercial PRRSV-1 MLV vaccine against a virulent PRRSV-1 strain based on the clinical outcome and virus replication was evaluated within the scope of our study. According to our results, the vaccination of gilts resulted in a significant reduction in the proportion of fetuses showing gross changes during necropsy. Under the conditions of this study, the fetuses of NV–I gilts were 23.3 times more likely to have an impaired fetal preservation status than fetuses from V–I gilts. Among non-viable fetuses, MEC was the most frequently encountered fetal preservation status. In addition, the highest viral load in the thymus was detected in MEC fetuses. Meconium staining of fetuses is caused by fetal stress and anorexia and is described as an early gross fetal condition occurring in PRRSV infection (67, 68). Fetuses with meconium-stained fluid on the face and body are likely to die within a few days (58). As termination of the study was done around gestation day 105, it can be speculated that the percentage of dead animals would have been even higher if pregnancy had remained until the normal term.

Maternal viremia serves as a prerequisite for PRRSV replication in the endometrium and subsequent fetal infection through the fetal placenta (32). However, previous studies have demonstrated that PRRSV-1 MLV vaccination is not always capable of preventing or reducing viremia in gilts and fetuses (64). Therefore, it is noteworthy that, in the present study, serum viral load AUC was significantly lower in V–I gilts compared to NV–I gilts. Thus, vaccination led to a reduction of viremia in terms of duration and magnitude. Since transplacental infection is more than likely a main route of virus transmission in a herd, it is important to note that, in our study, the use of MLV prevented transplacental PRRSV transmission in three out of four litters. The exact mechanism of transplacental viral transmission and fetal death is still poorly understood (32, 41, 56, 59). The results from previous studies indicated that PRRSV infection of the maternal–fetal interface and events occurring in the fetal compartment were critical for the fetal outcome (32, 46, 56, 69). Several authors have highlighted the importance of PRRSV RNA concentration in the maternal–fetal interface. In accordance with Ladinig et al. (41), the viral levels in the endometrium adjacent to each fetus were associated with the RNA concentration in fetal thymus, and virus was detected more frequently in endometrial samples than in fetal tissues. As previously observed (41), fetal death or fetal compromise was not observed in all fetuses with PRRSV detection in the respective endometrium sample. As PRRSV replication in the endometrium precedes fetal infection (32), it might also be speculated that the percentage of compromised piglets would have been higher if the gilts had farrowed at normal term. A recent study has highlighted that the replication of PRRSV in fetal thymus does not occur before 1 week post-infection (58).

The results from previous studies indicate that the concentration of PRRSV in non-reproductive systemic or lymphoid tissues have a minor impact on the odds of fetal death and fetal viral load (46). Interestingly, in the present study, the serum viral load AUC and viral concentration in the tonsils and uterine lymphoid tissue of the gilts were significantly associated with the proportion of PCR-positive fetuses (serum and/or thymus) per litter, but not viral load in gilt lung tissue and tracheobronchial lymph nodes. However, under the conditions of our study, gilt viral load seemed to have an important effect on the number of PRRSV-positive fetuses. This discrepancy between the two settings might be attributed not only to the different PRRSV strains used in the two experiments but also to the number of samples and parameters studied.

In conclusion, the high virulence of AUT15-33 could be confirmed phenotypically in an experimental reproductive model. The application of a commercially available modified live PRRSV vaccine showed promising results in reducing viremia and tissue viral load in gilts and their fetuses after an experimental infection with the virulent PRRSV-1 strain AUT15-33. In particular, the vaccine was able to prevent *in utero* PRRSV transmission to piglets in three out of four litters and to inhibit fetal compromise and fetal death.

## Data Availability Statement

The original contributions presented in the study are included in the article/Supplementary Material, further inquiries can be directed to the corresponding author/s.

## Ethics Statement

The animal study was reviewed and approved by the Institutional Ethics and Animal Welfare Committee (Vetmeduni Vienna) and the Austrian national authority according to §§ 26ff. of Animal Experiments Act, Tierversuchsgesetz 2012 – TVG 2012 (accession number: GZ 68.205/0142-WF/V/3b/2016).

## Author Contributions

HK, JS, TR, and AL wrote the manuscript and interpreted the results. HK, CK, and AL performed the study (recording of clinical assessment data and collection of blood). HK, JS, CK, ES, UR, EV, GB, and AL performed the necropsy and collection of samples. YZ conducted the statistical analyses. MZ and H-WC carried out the RT-qPCR. CR carried out the NGS analysis. TR was responsible for the overall virological investigations. AL and TR prepared the study protocol. AL supervised the overall project. All authors reviewed and contributed to the writing of the manuscript and approved the final version.

## Funding

The authors declare that this study received funding from Boehringer Ingelheim Vetmedica GmbH. The funder provided support in sample collection during necropsies, but was not involved in the study design, analysis, interpretation of data, the writing of this article or the decision to submit it for publication.

## Conflict of Interest

The authors declare that the research was conducted in the absence of any commercial or financial relationships that could be construed as a potential conflict of interest.

## Publisher's Note

All claims expressed in this article are solely those of the authors and do not necessarily represent those of their affiliated organizations, or those of the publisher, the editors and the reviewers. Any product that may be evaluated in this article, or claim that may be made by its manufacturer, is not guaranteed or endorsed by the publisher.

## Abbreviations

AUC: area under the curve
AUT: autolyzed
BSL: biosafety level
CRL: crown-rump length
Ct: cycle threshold
DEC: decomposed
dpi: days post-infection
emmeans: estimated marginal means
GE: genome equivalents
LM: linear model
LV: Lelystad virus
MEC: meconium-stained
MLV: modified live virus
NGS: next-generation sequencing
NV–I: non-vaccinated and infected
NV–NI: non-vaccinated and non-infected
NSP: nonstructural protein
nt: nucleotide
PAM: porcine alveolar macrophages
PBS: phosphate-buffered saline
PRRSV: porcine reproductive and respiratory syndrome virus
RT-qPCR: reverse transcription quantitative polymerase chain reaction
V–I: vaccinated and infected
V–NI: vaccinated and non-infected
VIA: viable.
